# Erratum

**DOI:** 10.1096/fba.1251

**Published:** 2021-06-03

In the article by Zhang et al.^1^ the authors report a composition error in the published article, the labels in Figure 1A were shifted in processing, overlapping the edge of the image.

The correct Figure 1 and legend are as follows.

**FIGURE 1:**
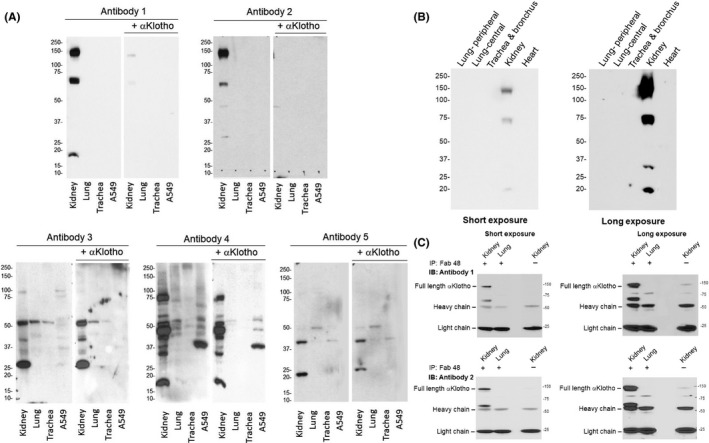
Immunoprecipitation‐immunoblot and for αKlotho in murine lung and A549 lung epithelial cells. Studies were performed with five antibodies. Antibody 1: Rat monoclonal Anti‐αKlotho Kl1 (KM2076). Antibody 2: Rat monoclonal Anti‐αKlotho Kl2 (KM2119). Antibody 3: R&D Cat# MAB1819; Antibody 4: Abcam Cat# ab203576; Antibody 5: Santa Cruz Cat# sc74205. Three wild‐type mice were used. (A), Representative immunoblot of lung lysate and A549 lung epithelial cell line probed with each antibody and blockade with purified recombinant αKlotho. Kidney lysate served as positive control. (B), Representative immunoblot of αKlotho in samples taken from different regions of the lung and probed using Antibody 1. Kidney served as positive control and the heart as negative control. (C), Representative immunoprecipitation (IP)‐immunoblot (IB) of αKlotho immunoprecipitated from lung lysate of two animals using a well validated synthetic anti‐αKlotho antibody sb48 (synonymous with sb106)6 and blotted with Antibodies 1 to 5. In (B) and (C), both a short and a long exposure are shown (left and right panels, respectively). Kidney lysate served as positive control
